# Supplementary material for: Maternal Body Mass Index Is Associated with Profile Variation in Circulating MicroRNAs at First Trimester of Pregnancy
Source: Biomedicines. 2022 Jul 18;10(7):1726. doi: 10.3390/biomedicines10071726 (PMC9313007; doi:10.3390/biomedicines10071726)
Supplement: Supplementary file 1 [file biomedicines-10-01726-s001.zip › Supplementary Figure S1.pdf]

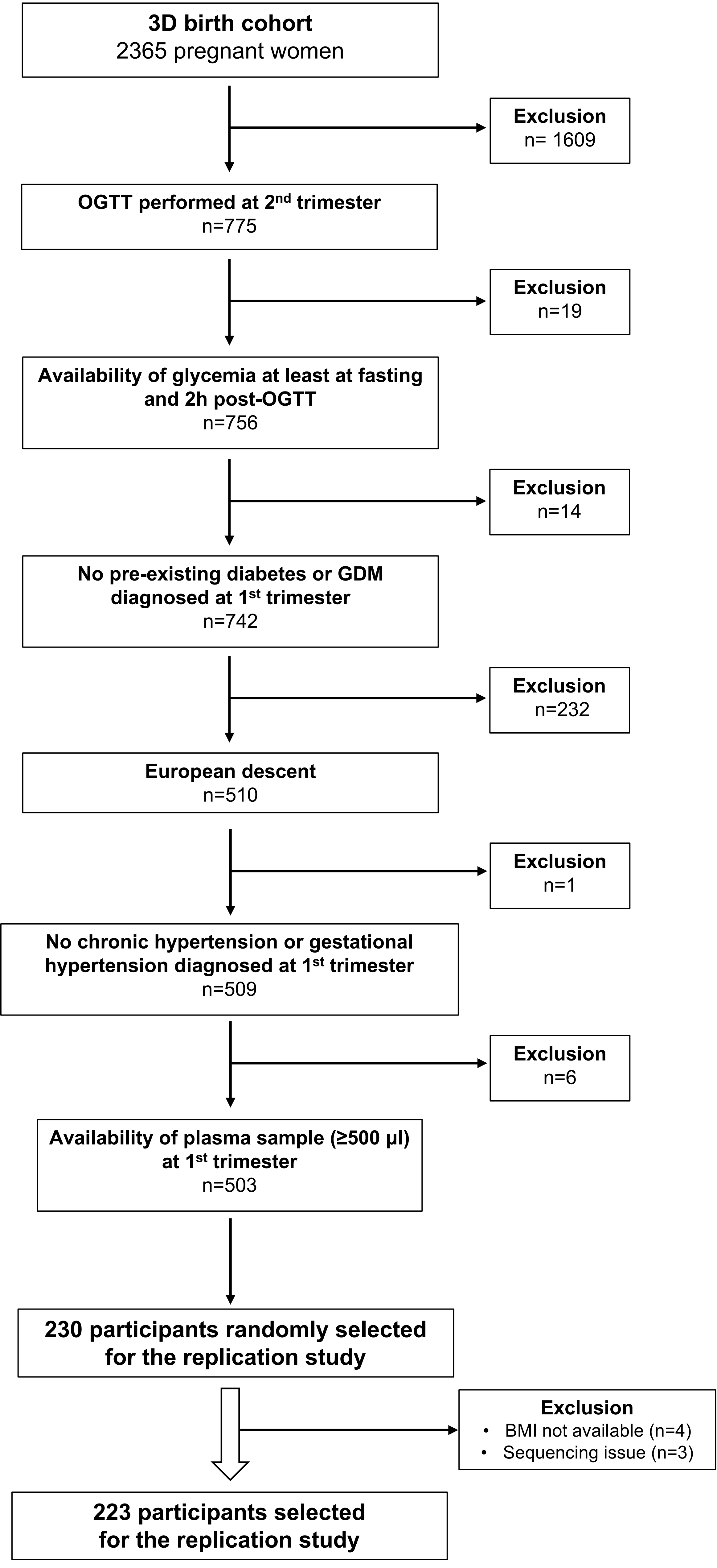
3D birth cohort
2365 pregnant women
Exclusion
n= 1609
OGTT performed at 2nd trimester
n=775
Exclusion
n=19
Availability of glycemia at least at fasting and 2h post-OGTT
n=756
Exclusion
n=14
No pre-existing diabetes or GDM diagnosed at 1st trimester
n=742
Exclusion
n=232
European descent
n=510
Exclusion
n=1
No chronic hypertension or gestational hypertension diagnosed at 1st trimester
n=509
Exclusion
n=6
Availability of plasma sample (≥500 µl) at 1st trimester
n=503
230 participants randomly selected for the replication study
Exclusion
BMI not available (n=4)
Sequencing issue (n=3)
223 participants selected for the replication study
