# Supplementary material for: Maternal Body Mass Index Is Associated with Profile Variation in Circulating MicroRNAs at First Trimester of Pregnancy
Source: Biomedicines. 2022 Jul 18;10(7):1726. doi: 10.3390/biomedicines10071726 (PMC9313007; doi:10.3390/biomedicines10071726)
Supplement: Supplementary file 1 [file biomedicines-10-01726-s001.zip › Supplementary Table S1.pdf]

Supplementary Table S1: List of miRNAs significantly associated with maternal BMI at the first trimester of pregnancy in the Gen3G cohort and replication results obtained in the 3D cohort for miRNAs significantly associated with maternal BMI at 1st trimester of pregnancy in the Gen3G cohort

	Gen3G				3D		
**miRNA**	**DESeq2 normalized read count (Mean ± SD)**	**Fold change***	**Unadjusted p-value**	**FDR-adjusted q-value**	**DESeq2 normalized read count (Mean ± SD)**	**Fold change***	**Unadjusted p-value**
hsa-miR-1323[a]	146.39 ± 230.60	0.957	2.79E-10	9.60E-08	676.45 ± 581.88	0.966	2.56E-05
hsa-miR-516b-5p[a]	101.50 ± 150.35	0.958	1.79E-10	9.60E-08	327.56 ± 247.26	0.966	2.92E-05
hsa-miR-371a-5p[c]	11.02 ± 17.09	0.941	2.13E-10	9.60E-08	28.15 ± 30.58	0.999	0.46859
hsa-miR-525-5p[a]	9.07 ± 15.31	0.952	1.63E-09	4.21E-07	79.89 ± 71.16	0.982	0.04471
hsa-miR-516a-5p[a]	31.25 ± 53.81	0.959	4.55E-09	7.83E-07	150.92 ± 135.76	0.974	0.00141
hsa-miR-524-5p[a]	8.64 ± 14.48	0.952	3.90E-09	7.83E-07	45.14 ± 41.69	0.991	0.17836
hsa-miR-518e-5p[a]	43.96 ± 62.52	0.962	8.92E-09	1.22E-06	99.07 ± 88.52	0.981	0.02118
hsa-miR-520a-3p[a]	86.75 ± 132.37	0.960	9.41E-09	1.22E-06	220.91 ± 243.90	0.982	0.04009
hsa-miR-518e-3p[a]	7.99 ± 14.22	0.956	2.02E-08	2.09E-06	29.60 ± 25.75	0.971	0.00189
hsa-miR-520d-5p[a]	5.92 ± 10.35	0.955	1.98E-08	2.09E-06	15.26 ± 16.99	0.993	0.29110
hsa-miR-512-3p[a]	287.04 ± 575.48	0.963	3.63E-08	3.41E-06	771.74 ± 982.11	0.982	0.02747
hsa-miR-1283[a]	57.10 ± 87.82	0.962	4.17E-08	3.59E-06	203.52 ± 173.84	0.977	0.00635
hsa-miR-517a-3p[a]	20.19 ± 37.78	0.960	5.17E-08	4.11E-06	76.56 ± 73.88	0.980	0.01599
hsa-miR-526b-5p[a]	17.12 ± 25.90	0.962	2.19E-07	1.62E-05	39.62 ± 34.83	0.977	0.01056
hsa-miR-373-3p[c]	19.85 ± 27.89	0.958	2.73E-07	1.88E-05	27.39 ± 52.26	1.002	0.43977
hsa-miR-517-5p[a]	16.90 ± 29.75	0.962	9.56E-07	6.17E-05	70.67 ± 67.45	0.981	0.02191
hsa-miR-515-3p[a]	3.13 ± 6.06	0.950	1.09E-06	6.60E-05	14.24 ± 18.36	0.980	0.06400
hsa-miR-1247-5p[b]	3.00 ± 4.54	0.952	2.81E-06	0.00016	12.21 ± 10.81	0.986	0.15508
hsa-miR-141-3p	147.17 ± 382.16	0.972	4.65E-06	0.00025	553.01 ± 632.80	1.016	0.03580

hsa-miR-519c-3p[a]	11.08 ± 20.08	0.965	5.11E-06	0.00026	36.44 ± 32.83	0.966	0.00034
hsa-miR-524-3p[a]	3.45 ± 6.77	0.953	6.56E-06	0.00032	12.17 ± 15.76	0.989	0.22380
hsa-miR-518d-5p[a]	3.31 ± 6.42	0.959	7.62E-06	0.00036	20.97 ± 17.86	0.994	0.28837
hsa-miR-505-3p	76.18 ± 58.08	1.012	1.31E-05	0.00059	139.92 ± 110.74	1.003	0.25405
hsa-miR-519d-5p[a]	6.12 ± 9.51	0.961	1.53E-05	0.00063	10.75 ± 11.77	0.969	0.01205
hsa-miR-515-5p[a]	10.50 ± 19.12	0.967	1.50E-05	0.00063	26.70 ± 26.08	0.968	0.00076
hsa-miR-629-5p	103.40 ± 68.36	0.984	2.40E-05	0.00096	211.04 ± 141.95	1.008	0.09565
hsa-miR-517c-3p[a]	3.30 ± 6.41	0.957	3.45E-05	0.00132	11.86 ± 13.92	1.002	0.43519
hsa-miR-205-5p	58.53 ± 84.88	0.975	4.02E-05	0.00148	157.07 ± 196.60	0.993	0.25806
hsa-miR-27b-3p	29699.76 ± 20347.78	1.008	4.98E-05	0.00172	55214.27 ± 41098.16	1.011	0.00110
hsa-miR-885-5p	13.50 ± 17.92	1.036	4.98E-05	0.00172	40.63 ± 78.98	1.034	0.00588
hsa-miR-483-5p	36.48 ± 39.39	0.976	5.61E-05	0.00187	104.49 ± 117.74	0.999	0.45703
hsa-miR-320c	165.01 ± 118.01	0.987	6.57E-05	0.00212	375.56 ± 308.74	1.010	0.06723
hsa-miR-372-3p[c]	3.70 ± 7.52	0.955	6.84E-05	0.00214	7.19 ± 11.22	0.992	0.33030
hsa-miR-200a-3p	29.32 ± 63.76	0.975	7.07E-05	0.00215	49.87 ± 55.29	1.000	0.48114
hsa-miR-518a-3p[a]	2.11 ± 4.62	0.954	9.82E-05	0.00290	7.62 ± 9.51	1.003	0.42395
hsa-miR-194-3p	2.69 ± 3.37	1.032	0.00018	0.00506	1.98 ± 3.68	1.001	0.48502
hsa-miR-519a-3p[a]	2.09 ± 3.98	0.959	0.00018	0.00512	8.20 ± 9.79	0.993	0.31122
hsa-miR-6884-5p	1.39 ± 2.04	0.952	0.00024	0.00653	2.17 ± 3.39	0.979	0.22718
hsa-miR-518f-5p[a]	2.63 ± 5.45	0.964	0.00025	0.00653	19.75 ± 16.93	0.999	0.44573
hsa-miR-520a-5p[a]	3.06 ± 5.66	0.963	0.00032	0.00830	27.63 ± 27.09	0.965	0.00136
hsa-miR-151b	526.36 ± 350.29	0.993	0.00037	0.00937	4.16 ± 6.00	1.019	0.12552
hsa-miR-375	1938.28 ± 3939.32	0.975	0.00040	0.00986	2900.08 ± 2878.58	0.982	0.03889
hsa-miR-518a-5p[a]	5.69 ± 9.41	0.971	0.00063	0.01512	16.55 ± 17.83	0.984	0.10443
hsa-miR-7977	41.69 ± 35.16	1.012	0.00066	0.01551	87.91 ± 99.41	0.999	0.42683

hsa-miR-122-5p	3282.18 ± 4306.34	1.028	0.00069	0.01589	4952.96 ± 7522.35	1.001	0.45267
hsa-miR-490-3p	6.37 ± 11.51	1.026	0.00071	0.01589	9.73 ± 18.17	0.998	0.43845
hsa-miR-320d	40.53 ± 37.85	0.986	0.00083	0.01820	100.05 ± 91.01	1.006	0.19941
hsa-miR-143-3p	31222.04 ± 25934.52	0.988	0.00087	0.01824	56150.60 ± 28383.73	0.987	0.08438
hsa-miR-7976	6.87 ± 7.72	0.978	0.00086	0.01824	23.65 ± 18.71	1.009	0.15976
hsa-miR-520d-3p[a]	9.80 ± 15.84	0.972	0.00091	0.01877	10.77 ± 13.37	0.966	0.00638
hsa-miR-574-3p	152.16 ± 109.54	1.009	0.00116	0.02332	361.20 ± 393.74	0.997	0.33673
hsa-miR-873-3p	4.36 ± 6.39	0.970	0.00117	0.02332	14.18 ± 14.84	0.986	0.15153
hsa-miR-429	13.50 ± 26.08	0.979	0.00134	0.02621	29.79 ± 33.02	1.014	0.08944
hsa-miR-592	1.64 ± 3.00	1.042	0.00162	0.03103	2.43 ± 4.89	1.058	0.00400
hsa-miR-155-5p	916.44 ± 519.38	1.006	0.00173	0.03203	598.09 ± 474.55	1.005	0.09079
hsa-miR-6819-3p	42.15 ± 32.51	0.986	0.00174	0.03203	97.97 ± 91.86	0.991	0.11547
hsa-miR-4286	70.98 ± 77.81	1.015	0.00187	0.03339	128.41 ± 150.47	1.002	0.39069
hsa-miR-193b-3p	4.13 ± 5.64	1.029	0.00187	0.03339	9.04 ± 10.98	1.014	0.13756
hsa-miR-21-5p	85256.17 ± 61108.55	1.005	0.00224	0.03811	82095.57 ± 63195.51	1.008	0.02738
hsa-miR-145-3p	29.39 ± 30.87	0.987	0.00225	0.03811	108.50 ± 87.26	0.993	0.14479
hsa-miR-520f-5p[a]	1.14 ± 2.38	0.954	0.00220	0.03811	2.10 ± 3.62	0.970	0.09179

Model adjusted for sequencing run and lane, gestational age, maternal age at the first trimester of pregnancy, and parity. * Fold changes represent the change in miRNA abundance for each increase of one unit of maternal BMI at 1st trimester of pregnancy. [a] miRNAs from C19MC, [b] miRNAs from C14MC and [c] miRNAs from miR-371-3 miRNAs cluster. Abbreviations: 3D: Design, Develop, Discover birth cohort; FDR: False discovery rate; Gen3G: Genetics of Glucose regulation in Gestation and Growth birth cohort; SD: Standard deviation.
